# Decision-Making Process for the Management of Acute Stroke in Patients on Oral Anticoagulant: From Guidelines to Clinical Routine

**DOI:** 10.3389/fneur.2021.794001

**Published:** 2022-01-05

**Authors:** Igor Sibon, Mikael Mazighi, Didier Smadja

**Affiliations:** ^1^Stroke Unit, Department of Neurology, Bordeaux University Hospital, Bordeaux, France; ^2^Department of Interventional Neuroradiology, Rothschild Foundation Hospital, University of Paris, Laboratory of Vascular Translational Sciences, Paris, France; ^3^Stroke Unit, INSERM U895, Department of Neurology, Centre Hospitalier Sud-Francilien, Paris-Saclay University, Corbeil-Essonnes, France

**Keywords:** stroke, haemorrhage, anticoagulant, idarucizumab, thrombolysis

## Abstract

**Background:** The occurrence of both ischaemic (IS) and haemorrhagic stroke in patients on anticoagulation is a major issue due to the frequency of their prescriptions in westernised countries and the expected impact of anticoagulant activity on recanalization during an IS or on the outcomes associated with intracerebral haemorrhage (ICH). Several guidelines are available but sometimes differ in their conclusions or regarding specific issues, and their application in routine emergency settings may be limited by particular individual issues or heterogeneous local specificities.

**Methods:** Based on the current guidelines and additional published data, the algorithms proposed in this paper aim to help the decision-making process regarding stroke management in the setting of concurrent anticoagulants by addressing specific clinical situations based on clinical variables commonly encountered in real-world practise.

**Results:** For patients on non–vitamin K oral anticoagulants, reversion can be achieved with specific antidotes, but only idarucizumab, the specific dabigatran antidote, is indicated in both IS and ICH. Due to the low risk of a prothrombotic effect, idarucizumab can be immediately used in IS patients eligible for thrombolysis before the dabigatran concentration is known. To optimise ICH management, the time since symptom onset, with thresholds proposed at 6 and 9 hours based on the expected timing of haematoma expansion, could also to be taken into account.

**Conclusions:** Anticoagulant reversal in patients presenting with a stroke remains a major issue, and algorithms based on a step-by-step approach may be useful for clinical practise. Real-life studies strongly support the benefits of idarucizumab availability in stroke units and emergency departments.

## Introduction

Oral anticoagulants are frequently used in westernised countries due to their indication in the management of cardio-embolic diseases, mainly in the prevention of stroke in patients with atrial fibrillation (AF) (1, 2). Vitamin K antagonists (VKA), like warfarin, have been widely prescribed over the past decades for preventing stroke, but their use is limited by a narrow therapeutic interval requiring frequent monitoring of anticoagulant activity by the assessment of the international normalised ratio (INR). More recently, non–vitamin K oral anticoagulants (NOACs), including the direct thrombin inhibitor dabigatran (3) and the factor Xa inhibitors apixaban (4), rivaroxaban (5), and edoxaban (6), have emerged as suitable alternatives, with a more favourable risk-benefit profile than warfarin (7), and are now considered the preferred choice for the prevention of stroke in patients with AF (2, 8).

Regarding ischemic stroke (IS) events in patients on anticoagulants, prior warfarin treatment has been shown to be associated with a lower initial severity and a lower risk of death or disability at 3 months (9). In clinical trials assessing NOACs in AF patients, IS and intracerebral haemorrhage (ICH) were found to occur in approximately 1 to 2% and 0.3 to 0.5% of patients per year, respectively (3–6). Moreover, IS in AF patients on NOACs is associated with a smaller infarct volume and a decreased risk of greater proximal artery occlusion than in a situation with no anticoagulation (10), but it mainly corresponds to a small vessel disease consistent with a mechanism other than that of AF-induced IS (11).

Specific reversal agents have been developed for NOACs allowing quick and durable neutralisation of the anticoagulant effect (12, 13). In this context, guidelines published by local or international societies define the most appropriate strategy for stroke management in patients on anticoagulants (2, 8, 14, 15). While those guidelines are to be used as references to guide the decision-making process, their conclusion may significantly differ (16), and their application may be dependent on individual contexts and local specificities regarding, for instance, the delay before the results of specific anticoagulant blood tests are available or the availability of specific reversal agents. Furthermore, some particular issues, like the delay of ICH-associated symptom onset, may have to be considered according to a case-by-case approach.

Based on current guidelines and the most recent publications addressing IS and ICH management in patients on anticoagulants, this paper aims to provide step-by-step decision trees that take alternate options into consideration at each step of the decision process according to the heterogeneity of a real-life setting. The options proposed also refer to series, case reports, and opinion papers addressing specific issues of stroke management that may be helpful in routine practise. Publications were selected from PubMed database by using “ischemic stroke” OR “intracerebral haemorrhage” AND “anticoagulants” OR “anticoagulation reversal” as keywords, with a focus on the past five years in order to take into consideration the possible use of reversal agents. Regarding guidelines from Societies and Associations concerned with the topics, the last updated versions were taken into consideration. A state of the art of the expected benefits of reversal agents is proposed beforehand.

## Reversion of Anticoagulant Activity: What Are We Talking About?

### Non-specific Anticoagulation Reversal

Fresh frozen plasma (FFP) has been widely used for many years, but its use is limited in routine practise by blood type matching, maximal acceptable or possible complications related to fluid overload, so its use has been supplanted by prothrombin complex concentrates (PCCs), particularly in situations involving critical bleeding (17, 18). Four-factor PCC consists of vitamin K-dependent coagulation factors (II, VII, IX, and X) with a final overall clotting factor concentration that is approximately 25 times higher than in normal plasma (19). Several studies have shown that PCCs act more quickly and effectively than FFP in terms of correcting the INR of patients on VKA (16, 18). The effect of PCC on NOAC-associated ICH is less clear. While haemostasis is expected to be effective in two-thirds of patients (20–22), a retrospective study of patients with NOAC-related ICH showed that PCC administration prior to imaging-based follow-up was not significantly associated with a reduced rate of hematoma expansion (HE) and had no effect on mortality and functional outcome at discharge or at 3 months (23). This study points out the need for specific reversal agents in cases of ICH on NOACs.

In clinical situations that warrant a rapid reduction in NOAC exposure, like ICH, charcoal may be used to adsorb drugs present in the gastrointestinal tract and then reduce their bioavailability, as well as enhance elimination by interrupting the enteroenteric recycling (24, 25). Charcoal is recommended in cases of recent ingestion of a NOAC (<4 h) in contexts of overdose, as it reduces absorption (8), or in cases of ICH (2, 13). Haemodialysis is an additional option for dabigatran, but modelling studies demonstrated that idarucizumab administration results in a complete and immediate reduction in dabigatran plasma concentrations, whereas a 4-h haemodialysis reduces dabigatran plasma concentrations by approximately 60% (26). Therefore, this strategy should be discussed in the context of life-threatening haemorrhage only when a specific reversal agent such as idarucizumab is unavailable (13).

### Specific Anticoagulation Reversal

#### Vitamin K

While being considered as a natural VKA reversal agent, vitamin K alone does not allow a rapid restoration of coagulation (estimated to occur within 4 to 6 h with intravenous vitamin K), which is required in cases of major or life-threatening bleeding (27), so its use is recommended in addition to fast reversal strategies, including PCC, to prevent a re-increase in INR (14).

#### Idarucizumab

Idarucizumab, the dabigatran antidote, is a humanised monoclonal antibody fragment that binds dabigatran with high affinity (350 times stronger than its affinity for thrombin) and specificity (28). The Reversal Effects of Idarucizumab on Active Dabigatran (RE-VERSE AD) multicentre, prospective, single cohort study, which included 503 patients with uncontrollable or life-threatening bleeding, showed a 100% reversal (assessed by the diluted thrombin time) within 4 h after the administration of two 50-ml bolus intravenous infusions of idarucizumab (2 ×2.5 g) that were no more than 15 min apart (29). The median time to haemostasis was 2.5 h (95% CI, 2.2 to 3.9 h), and reversal occurred independently of renal function or dabigatran concentration at baseline (median concentration around 100 ng/mL, with an upper range above 400 ng/mL). A recurrent increase in dabigatran level (>20 ng/mL) was observed after 12 h in 23% of patients, which was associated with recurrence or continuous bleeding in 10 patients with uncontrollable bleeding. Thrombotic events at 90 days were reported in around 7% of patients, and there were no serious adverse safety signals, with most of the events being a worsening of the index event or a coexisting condition. Finally, the 30-day mortality rate was around 13%, which is three times lower than the rate reported in patients presenting with ICH while being on oral anticoagulants in the absence of antidote (30).

#### Andexanet Alfa

Regarding the specific reversal of factor Xa inhibitors, andexanet alfa allows activated factor X to convert prothrombin to thrombin and then restore coagulation (31, 32). The ANNEXA-4 study assessed the efficacy of andexanet alfa as a bolus during a period of 15 to 30 min, followed by a 2-h infusion, in 47 patients presenting with acute major bleeding within 18 h after administration of factor-Xa inhibitors (33). The median anti-factor Xa activity was decreased by 89 and 93% in patients receiving rivaroxaban and apixaban, respectively. The corresponding concentrations were around 30 and 12 ng/mL during the 2-h infusion but increased at 4 h to reach more than 100 ng/mL and remained in this same range at 8 and 12 h. The safety assessment of 67 patients showed that the rates of thrombotic events (mainly stroke and deep vein thrombosis) and mortality were 18 and 15%, respectively. While andexanet alfa has been approved by the Food and Drug Administration (FDA), the possible thrombotic effect prompted the FDA to include a warning box in the prescription information. Andexanet alfa has received conditional marketing authorisation from the European Medical Agency (34) and remains unapproved in several countries. Finally, and importantly, commercial assays of anti-factor Xa activity following administration of andexanet alfa are unsuitable, as these assays result in erroneously elevated anti-factor Xa activity levels, thereby causing a substantial underestimation of the reversal activity of andexanet alfa (34).

## Management of IS In Patients on Anticoagulants

Intravenous thrombolysis (IVT) consists of the administration of recombinant tissue plasminogen activator (rt-PA), mainly alteplase, although data are now available with tenecteplase, a genetically modified form of alteplase with a higher fibrin specificity and longer half-life (35, 36). Regarding patients presenting with IS and salvageable brain tissue, the multicentre, randomised, placebo-controlled EXTEND trial indicated that thrombolysis performed in patients who had a favourable perfusion-imaging profile between 4.5 and 9.0 h after stroke onset resulted in a higher percentage of patients with no or minor neurologic deficits (37). In patients who have had a stroke with an unknown time of onset with a diffusion-weighted imaging-fluid-attenuated inversion recovery (DWI-FLAIR) or perfusion mismatch, IVT has been shown to result in a better functional outcome at 90 days than placebo or standard care (38).

The updated guidelines jointly published by the American Heart (AHA) and Stroke (ASA) Associations state that endovascular mechanical thrombectomy (MT), represents the gold standard in the management of acute IS due to large vessel occlusion (39). Anticoagulant medication adds an extra layer of complexity in the management of patients suffering acute IS. In addition to common parameters such as clinical severity, stroke volume and delays from symptoms onset, acute therapy is influenced by access to specific reversal agents, other available reperfusion strategies, and measured anticoagulant activity.

### Recommendation for IVT

**Figure 1:**
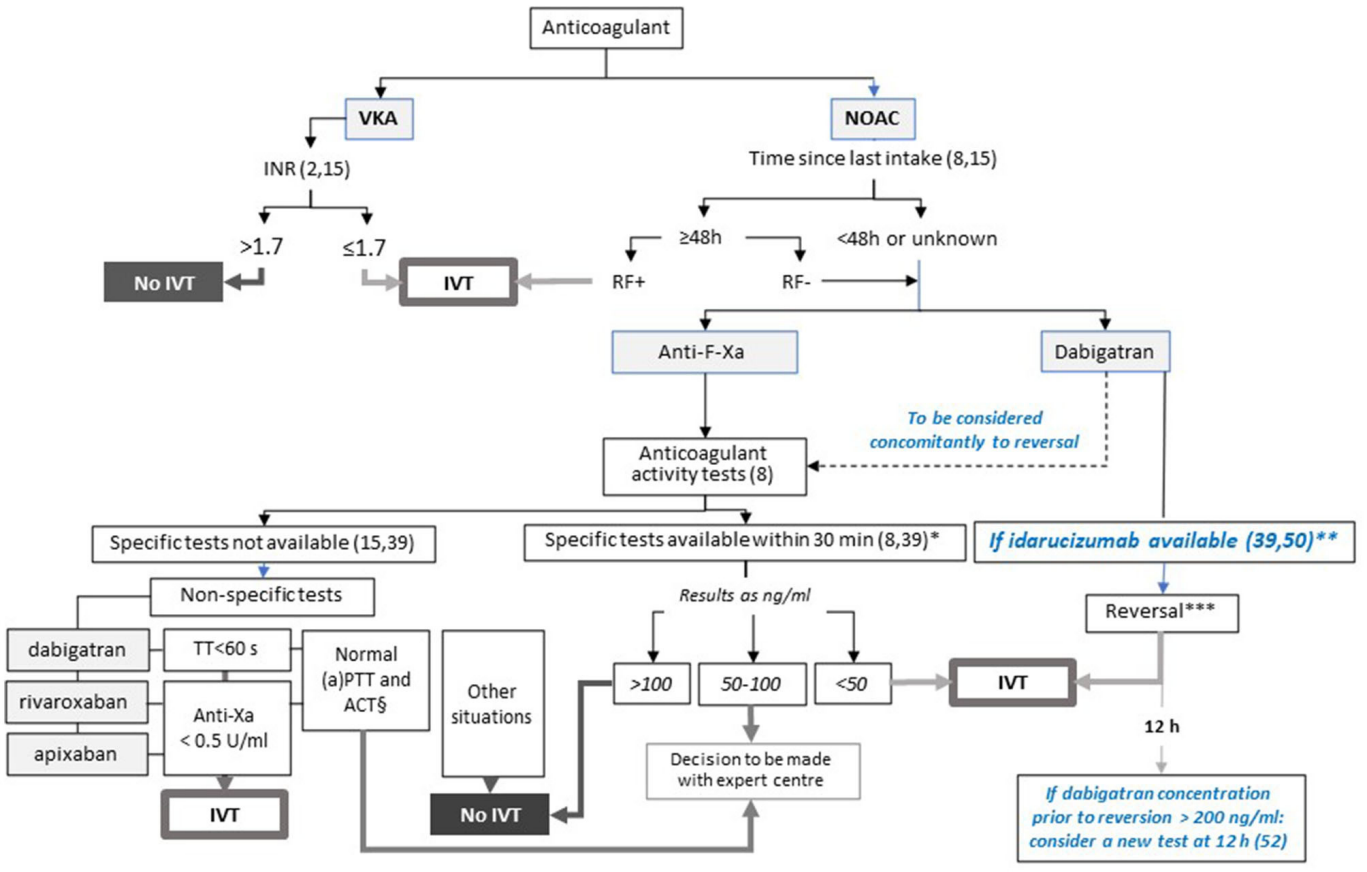
Proposed algorithm for the management of ischemic stroke in patients eligible for IVT according to the anticoagulant, and local specificities. This figure presents the decision-making process for IVT in patients presenting with IS. In case of proximal and large vessel occlusion, MT should be considered alone or coupled with IVT. Steps in blue are based on data from the literature and are not mentioned in the current guidelines. *Based on usual practise. **Consider dabigatran dosing if a high concentration is suspected. *** To be undertaken before dabigatran concentration is known. In case of high concentration of dabigatran (>200 ng/ml) prior to reversal, consider assessment of anticoagulation activity 12h after reversal and, if dabigatran concentration > 50 ng/ml, a second administration of idarucizumab. § For dabigatran and rivaroxaban. ACT, activated cephalin time; aPTT: activated partial thromboplastin time; IVT: intravenous thrombolysis; RF+/RF-: normal (creatinine clearance ≥50 ml/min) or abnormal renal function according to the last dosage available; TT: thrombin time.

IVT should not be administered in patients on a full anticoagulation dose (15, 39). Regarding endovascular MT, a retrospective analysis of a cohort of patients presenting with IS while being on NOAC or VKA showed that intra-vascular therapy was safe, with patients achieving similar rates of good angiographic and clinical outcomes as with normal haemostasis and IVT (40). MT should be considered alone or coupled with IVT if there is a target vessel occlusion and if the procedure is indicated and feasible (8). Thus, the main challenge regarding the management of IS in patients on oral anticoagulants is the assessment of the anticoagulation activity and, when possible, its reversion. The step-by-step approach proposed below and illustrated in Figure 1 is primarily based on the current European Heart Rhythm Association (EHRA) and AHA/ASA guidelines (8, 15, 39). It also takes into consideration several studies and opinions like that jointly stated by the French Neurovascular Society and the French Study Group on Haemostasis and Thrombosis (41) in order to cover potential local heterogeneities.

### IVT in Patients on VKA

A pilot study that included consecutive IS patients on VKA showed that IVT carried out immediately after anticoagulation reversal by 4-factor PCC and vitamin K could be feasible and safe when the INR is above 1.7 (42). While encouraging, these results were obtained from a small sample of patients in the absence of a comparison group and require confirmation by further well-designed randomised controlled trials. In this context, IVT has to be considered in the management of IS occurring in patients on VKA for INR values of 1.7 or less, as retained in the guidelines of the European Society of Cardiology (ESC) (2) and of the European Stroke Organisation (ESO) (15). Point of care (POC) testing of the INR is not recommended to date by European and American guidelines before intravenous thrombolysis for assessing VKA activity. One study suggested the comparability of POC and central laboratory INR measurements (43). However, some discrepancies between these two evaluations still limit their usefulness in clinical practise and we would not recommend IVT for patients with INR > 1.2 measured with a POC.

### IVT in Patients on NOACs

A meta-analysis based on six studies and a total of 52,823 patients with IS suggests that the risk of symptomatic haemorrhagic transformation after IVT is not increased in patients given NOACs when compared with patients taking warfarin and with an INR of less than 1.7, or with those who did not take any anticoagulation, whatever the elapsed time between the last NOAC intake (less or more than 48 h). It also highlights inadequate data regarding the exact time of the last NOAC intake to be considered (44). On the basis of the half-lives of the available NOACs, which are estimated to be less than 18 h (16), the EHRA guidelines recommend IVT if the delay is 48 h or more and if the renal function is normal (creatinine clearance > 50 mL/min) (8, 15). Indeed, renal failure is expected to increase the exposure to the drug and then the risk of bleeding. When the time from the last intake is less than 48 h or is unknown or in situations of renal insufficiency, the strategy will depend on the NOAC considered with the option of an immediate reversion by idarucizumab in patients on dabigatran or the previous assessment of anticoagulant activity in patients on factor-Xa inhibitors or when idarucizumab is unavailable.

### Reversion by Idarucizumab in Patients on Dabigatran

Several case reports and series involving patients presenting with IS have shown that neutralisation of dabigatran activity by the immediate administration of idarucizumab made IVT possible in real-life practise in situations of common contra-indication of IVT on the basis of baseline dabigatran concentration (35, 36, 46–50). A systematic review coupled with a case series analysis indicates that in a context of IS with the last dabigatran intake within 24 h, idarucizumab administration before IVT is safe, with side effects being related to the stroke or to patients' characteristics rather than to the reversal of anticoagulation with idarucizumab (35).

Situations in which the period between the last intake of dabigatran and the admission is unknown are a major issue. Some authors suggest that in this context, the use of idarucizumab depends on the results of tests assessing anticoagulant activity (51), so idarucizumab is not administered to patients in the absence of significant anticoagulant activity. As previously highlighted, the effect of idarucizumab is not influenced by the baseline concentration of dabigatran, and the prothrombotic risk due to idarucizumab is low if there is any (29, 52). Retrospective data collected from German neurological/neurosurgical departments administering idarucizumab to all patients presenting with IS while being on dabigatran showed that administering IVT within 4.5 h of IS onset improved median NIHSS by 7 points in 78% of the patients, with no reported bleeding complications (53). In this cohort, activated partial thromboplastin time (aPTT) values were normal in 50% of cases, which indicates that in the absence of reversion, half of the patients would have been excluded from IVT, and supports the concept that reversion by idarucizumab does not have to be delayed by the availability of coagulation tests. This situation may enhance the chance for a good outcome, as illustrated by the case of a patient on dabigatran who was admitted for an acute IS and received idarucizumab and subsequently IVT, which resulted in a reduced door-to-needle time (54). In accordance with the French guidelines stating the possibility of administering idarucizumab whilst dabigatran concentration is low or zero (41), our position is to consider reversion by idarucizumab before the results of dabigatran activity are known so IVT can be started as soon as possible. If the tests reveal active concentrations of dabigatran prior to reversion, we suggest considering a further assessment of dabigatran activity after reversal and a re-administration of idarucizumab if the dabigatran concentration is higher than 50 ng/mL, a concentration under which no risk of haemorrhage induced by the anticoagulant activity is expected (41). The possibility of rebound effect of plasma dabigatran after reversal with idarucizumab, although corresponding to a rare situation mainly reported from isolated cases (55), has to be taken into consideration. An analysis of the published and original cases addressing situations in which there is a rebound after reversal of dabigatran with idarucizumab showed that an initial dabigatran plasma concentration above 200 ng/mL could discriminate patients with a rebound risk (55). Additionally, rebounds were mainly observed within 12 h after the administration of idarucizumab. Thus, while the cut-off of 200 ng/mL has to be prospectively confirmed, we propose retaining this value to consider whether the re-assessment of dabigatran activity is needed or not. Finally, based on the current studies comparing direct endovascular therapy (EVT) and EVT + IVT that are closed to reaching the non-inferiority margin, we propose to proceed directly to thrombectomy without reversal of anticoagulation for patients admitted directly to a comprehensive stroke-centre for an IS related to a proximal occlusion (56–58). However, for those managed according to a drip-and ship paradigm our position is to consider IVT following reversal of anticoagulation prior transferring the patient for EVT.

### Assessment of Anticoagulant Activity of NOACs

Coagulation tests have to be considered prior to IVT when the intake of NOAC has occurred within 48 h or is unknown (8, 15). It is recommended that blood levels of NOACs be given within a short delay, and the period of 30 min may be retained (8, 44). Specific NOAC tests should be preferred over non-specific tests, as they provide a more accurate level of anticoagulant activity. Based on expert consensus (59), the EHRA guidelines proposed a threshold of less than 30 ng/mL to consider IVT (8). More recently, based on the evaluation of the relation between post-IVT outcomes (mainly ICH) and baseline NOAC concentrations assessed by specific tests in patients presenting with acute IS and suspected use of NOAC within 48 h prior to hospital admission, a prospective study suggested that levels less than 50 ng/mL may be supportive for IVT, while patients with NOAC levels higher than 100 ng/mL should be excluded from IVT (60). On the basis of these results, we propose considering these thresholds, which are also retained by the French Neurovascular Society (41).

NOAC concentrations between 50 and 100 ng/mL then correspond to a grey zone and require referral to an expert centre in order to accurately define the individual benefit-risk ratio of IVT (61). When specific NOAC tests are not available during the timeframes, it is recommended to refer to routine non-specific tests. These tests include anti-Xa activity (threshold: 0.5 U/mL) for factor-Xa inhibitors. Thrombin time (threshold: 60 sec) for dabigatran and, alternately for rivaroxaban and dabigatran only, the normal vs. abnormal values of activated clotting time and prothrombin time may be considered. However, those tests are not reliable for an accurate assessment of NOAC activity (8) or remain to be validated in prospective studies (62), so specific testing should be recommended.

In cases in which the last intake of anti-factor Xa occurred in the past 2–4 h, the relation between the biological activity and the anticoagulant-induced risk of haemorrhage due to the persistent absorption of the anticoagulant remains to be clarified, as well as the indication of IVT in such situations.

## Management of ICH In Patients on Anticoagulants

**Figure 2:**
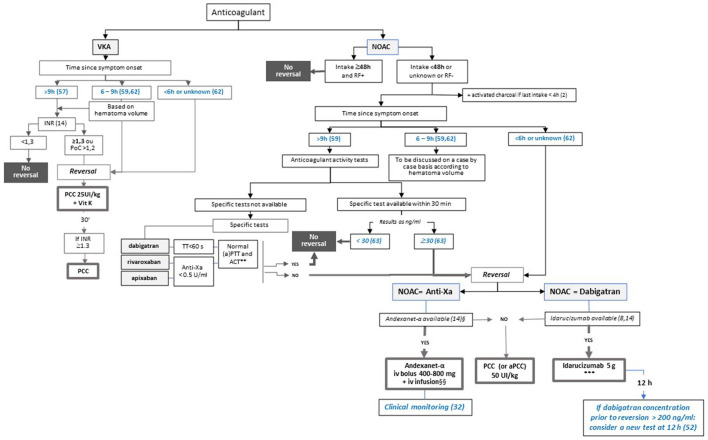
Proposed algorithm for the management of intracerebral haemorrhage according to the anticoagulant, time since symptom onset, and local specificities. This figure presents the decision-making process for anticoagulant reversal in patients presenting with HIC in taking into account thresholds for the time since symptom onset according to Al-Shahi Salman and colleagues, 2018 (65) and to Kuramatsu and colleagues, 2015 (68). Steps in blue are based on data from the literature and are not mentioned in the current guidelines. *Dose to be determined according to INR. **For dabigatran and rivaroxaban. ***In case of high concentration of dabigatran (>200 ng/ml) prior to reversal, consider assessment of anticoagulation activity 12h after reversal and, if dabigatran concentration > 50 ng/ml, a second administration of idarucizumab. § Approved for apixaban and rivaroxaban. §§ In case of high concentration of apixaban or rivaroxaban (>200 ng/ml) prior to reversal, consider assessment of anticoagulation activity according to clinical parameters. ACT: activated cephalin time; aPTT: activated partial thromboplastin time; IVT: intravenous thrombolysis; PCC: prothrombin complex concentrates; RF+/RF-: normal (creatinine clearance ≥50 ml/min) or abnormal renal function according to the last dosage available; TT: thrombin time.

ICH occurring in patients on anticoagulants is associated with a poorer prognosis, mainly due to a larger initial volume (63, 64) and a threefold increase in ICH expansion (OR 2.91, 95% CI 1.97–4.26) (65). Recent studies suggest that ICH is less severe when occurring on NOACs than on VKA, with a lower initial volume and a lower risk of expansion (64), but functional outcomes and mortality rate due to major bleeding are not expected to be different (66, 67). The acute management of ICH in patients on anticoagulants is based on admission in the neurovascular unit, a rapid lowering of systolic blood pressure to less than 140 mmHg, and reversion of anticoagulant treatment in order to limit HE (8, 14). The strategy of reversion depends on the anticoagulant considered, the time since the onset of symptoms, and the anticoagulant activity, and it may consist of specific or non-specific reversion (Figure 2).

The predicted probability of intracerebral HE is expected to rapidly decline within 0.5–3 h and to reach a plateau at 9 h (65). On the other hand, a logistic regression model using generalised estimating equations suggests that HE is no longer prevented by INR reversal after 5–6 h (68). On this basis, the algorithm for ICH management proposes that reversion is to be systematically considered if the time since symptom onset is 6 h or less and that anticoagulant activity is to be checked if the time is 9 h or more (Figure 2). The decision to be made in the grey zone of the occurrence of symptoms between 6 and 9 h should be based on expert decision.

### Reversion in Patients on VKA

In patients treated with VKA with an INR above normal, the ESO recommends fast reversion with PCC and the addition of vitamin K in order to prevent a re-increase in INR and to decrease HE and mortality (14). The INR threshold proposed in those guidelines is 1.3, which is different from the value of 1.2 proposed by Steiner and colleagues (69) on the basis of the threshold used in the INCH study (70), which stresses the fact that both optimal INR thresholds for treatment and to be targeted are yet to be defined (64). In a retrospective cohort study of ICH management that included 853 patients for analysis of HE reversal, an INR value of less than 1.3 showed the strongest positive association to prevent HE (area under the curve, 0.636; 95% CI, 0.596–0.676; *P* < 0.001) (67). Furthermore, a pooled analysis of ICH data from 16 stroke registries suggested that was associated with the lowest case fatality following ICH occurring on VKA treatment with an INR of 3 or more (71). Thus, we propose considering reversion in patients with an INR of at least 1.3 according to the ESO guidelines.

### Reversion in Patients on NOACs

Reversion should be considered if the time since the last intake is within 48 h or is unknown, or if renal function is abnormal. In situations in which there has been a recent NOAC intake (<4 h), oral activated charcoal is expected to reduce drug absorption and exposure and, while not retained in the last ESO guidelines (14), may be considered as an option when NOAC intake occurred within the past 4 h (12, 13).

Reversal of NOAC activity has to be systematically considered if the symptoms occurred within 6 h. According to the updated ESO guidelines, idarucizumab is strongly recommended for reversing the activity of dabigatran (14). Andexanet alfa is now considered as a possible option for patients on apixaban or rivaroxaban, but with a weak level of recommendation (14) (Figure 2). If specific reversal agents are not available, the use of 4-factor PCC or aPCC (37.5–50 IU/kg) is recommended (8, 14). However, their efficacy remains uncertain in regard to the reduction of HE, survival, and improvement of functional prognosis (23), which highlights the importance of the stroke units having access to NOAC antidotes.

To date, the association between NOAC activity and the risk of HE remains to be defined (72), and there is no recommendation concerning the use of specific biological dosages in the therapeutic management of ICH in patients on NOAC based on the time since symptom onset and anticoagulation activity. The algorithm proposed herein (Figure 2) considers that when the time since the symptom onset is 9 h or more, anticoagulation activity has to be assessed with specific (preferred) or non-specific tests (with the same method and thresholds as those described above for IS management), based on the capacity of the stroke unit. The French guidelines that focused on dabigatran reversion by idarucizumab in the management of bleeding and emergency invasive procedures retained the thresholds of 50 ng/mL and 30 ng/mL to define the persistence of anticoagulation activity and the necessity of reversion to enable the management of haemorrhage or an invasive procedure with a high risk of bleeding, respectively (73). In a review focusing on the management of ICH in patients receiving oral anticoagulants, Steiner and colleagues proposed considering reversal if the dabigatran concentration exceeds 30 ng/mL, which corresponds to the lower limit of quantification of the assay (69). We propose considering a threshold of 30 ng/mL to guide reversion so that a larger number of patients may benefit from this option.

Regarding specific reversal by dabigatran before the results of the tests are known, similarly to what is proposed in the management of IS, we consider that an elevated initial dabigatran concentration (> 200 ng/mL) warrants a second dosage 12 h after idarucizumab administration and a further administration of idarucizumab if the dabigatran concentration remains higher than 50 ng/mL. Concerning reversion of apixaban or rivaroxaban by andexanet alfa, and due to the unsuitability of available anti-factor Xa activity assays, treatment monitoring should be based on clinical parameters indicative of appropriate response (i.e., achievement of haemostasis), lack of efficacy (i.e., re-bleeding), and/or adverse events (i.e., thromboembolic events) (34). As of now, while the recommendation of the ESO for the use of idarucizumab in patients on dabigatran is strong, the recommendation of andexanet alfa in patients on rivaroxaban or apixaban is weak due to a lack of solid data in ICH and uncertainty as to whether the haemostatic effect outweighs the undesirable effects (14).

In situations in which symptom onset occurs between 6 and 9 h, the decision to reverse should be guided by expert centres on the basis of patients' thrombotic and haemorrhagic risks.

Finally, for patients admitted more than 9 h after symptoms onset, coagulation tests should be ordered urgently after admission, and anticoagulant will be reversed if modest anticoagulant activity is detected (see Figure 2).

## Perspectives

The management of acute strokes in patients on anticoagulants remains a major challenge in real clinical settings. When considering a step-by-step approach that takes account of particular contexts and/or local specificities, some options to be considered in emergency situations are not systematically and precisely defined in current guidelines regarding, for instance, the timing to be considered since the onset of symptom or the thresholds to retain for anticoagulation activity. Furthermore, the accumulation of data regarding specific NOAC antidotes justifies some proposals regarding the timing of reversal in emergencies. The present paper proposes two algorithms based on the current guidelines and completed with published data to take every possible option into consideration.

Regarding the immediate antidote-based reversion of anticoagulant activity in patients presenting with a stroke while on dabigatran, the possibility of administering idarucizumab whilst the drug level is low or zero (41) suggests that reversal of dabigatran before laboratory and imaging results are known may be considered prior to in-hospital admission. The administration of idarucizumab in the Melbourne mobile stroke unit (MSU) prior to IVT has been reported in three patients presenting with IS after dabigatran intake within the past 12 h (50). The mean time between idarucizumab administration and IVT was 10 min, and two patients subsequently underwent MT. Results at 24 h showed a small amount of asymptomatic petechial haemorrhage in one patient, and all patients demonstrated substantial neurological recovery and were discharged to inpatient rehabilitation. While performed on patients with substantial dabigatran activity as suggested by the delay since the last intake, rapid treatment with pre-hospital administration of idarucizumab prior to IVT was shown to be feasible and to facilitate hyperacute treatment. The feasibility of pre-hospital administration of idarucizumab is also supported by the reported case of an 82-year-old woman treated with dabigatran for AF who developed an acute subdural haematoma revealed by computed tomography of the brain performed in the MSU (74). The patient received idarucizumab prior to admission to the neurosurgery service. A CT scan 72 h later demonstrated a stable subdural haematoma, and following burr hole trephination, the patient had no focal neurological deficits and was discharged to a rehabilitation facility. While based on few cases, these data strongly support the importance of the availability and use of idarucizumab in MSUs to optimise early management of ICH and an early reversal prior to IVT in cases of IS, and advocate for further larger studies that include patients with possible low dabigatran concentrations.

Another major challenge is the management of the post-stroke phase. Available studies and current guidelines support an early reintroduction of anticoagulants after an IS, with a timing that remains to be defined and that should take into account thrombotic and haemorrhagic risks on the basis of stroke severity assessment by imaging. Resumption after an ICH should be considered after at least 4 weeks on an individual basis and with a preference for NOACs over VKA, as suggested by the ESC current guidelines (2). One key issue to keep in mind in clinical practise is the cause of stroke episodes occurring in patients on anticoagulants, which includes the possibility of underdosing. The percentage of patients being underdosed while not adhering to the criteria of dose reduction could reach 72% in “real-life” settings (11). An analysis based on a large U.S. database that included nearly 15,000 AF patients treated in routine clinical practise showed that in cases in which patients were treated with apixaban, underdosing was associated with a nearly fivefold increase in the risk of stroke (75). While strokes may occur independently of the use of NOACs, the choice and adjustment of the appropriate dose based on the patient's status and evolution is a major issue that specialists (cardiologists, neurologists) should be aware of.

In conclusion, the management of stroke occurring in patients on oral anticoagulants is an important issue due to the negative impact of anticoagulation on HE or on thrombolysis in cases of IS. While updated guidelines are available to help with decision making, there is a potential heterogeneity across stroke units regarding the availability of tests, antidotes, and recommended procedures. For this reason, algorithms that take several options into account according to a step-by-step approach, like those proposed herein, are expected to be useful for the management of ICH and IS, particularly concerning the key step of anticoagulation reversal. Given the proportion of patients treated with dabigatran for AF, the availability of idarucizumab in stroke units, including MSUs, should be encouraged, as it is expected to allow for an immediate reversal of dabigatran activity independently of its baseline concentration and then to optimise the outcomes of patients with ICH or eligible to IVT.

## Author Contributions

All authors had the idea for the article, and performed the literature search and data analysis, critically revised the work, and read and approved the final manuscript. The first draft of the manuscript was written by IS and all authors commented on previous versions of the manuscript.

## Conflict of Interest

IS received fees for editorial activities with Elsevier, served as advisor for Servier and Boehringer Ingelheim, received teaching honoraria from Medtronic, BMS-Pfizer, AstraZeneca and Bayer, as well as research support from the University Hospital of Bordeaux and the French Health Ministry. MM received speaker's honoraria from Boehringer Ingelheim and consulting honoraria from Boehringer Ingelheim, Acticor Biotech, Air Liquide and Amgen. DS received speaker's honoraria and consulting honoraria from Boehringer Ingelheim.

## Publisher's Note

All claims expressed in this article are solely those of the authors and do not necessarily represent those of their affiliated organizations, or those of the publisher, the editors and the reviewers. Any product that may be evaluated in this article, or claim that may be made by its manufacturer, is not guaranteed or endorsed by the publisher.
